# Efficacy of laryngeal mask epinephrine in neonatal resuscitation; an ovine study

**DOI:** 10.1038/s41390-025-04070-5

**Published:** 2025-05-12

**Authors:** Hamza Abbasi, Clariss Blanco, Arun Prasath, Mary Kasu, Sylvia Gugino, Justin Helman, Nicole Bradley, Lori Nielsen, Rocco A. Paluch, Praveen Chandrasekharan, Munmun Rawat

**Affiliations:** 1 John R. Oishei Children’s Hospital, Neonatology Department, Buffalo, NY USA; 2 State University of New York at Buffalo, Buffalo, NY USA; 3 NYC Health and Hospitals/Harlem, New York, NY USA; 4 University of Texas Southwestern, Dallas, TX USA; 5 University of Maryland Medical Center, Baltimore, MD USA

## Abstract

**Background:**

The efficacy of Laryngeal Mask Airway (LMA) epinephrine during neonatal resuscitation has not been studied. We hypothesize that LMA epinephrine is as effective as endotracheal tube (ETT) epinephrine.

**Methods:**

Sixteen fetal lambs were randomized in ETT or LMA group for ventilation and airway epinephrine administration after cord occlusion to induce complete cardiac arrest. Lambs were delivered and instrumented to continuously record pulmonary and systemic hemodynamics. After 5 min of cardiac arrest, lambs were resuscitated per NRP guidelines. Blood gases and plasma epinephrine levels were regularly measured during resuscitation.

**Results:**

Baseline characteristics were similar between the two groups. Incidence of return of spontaneous circulation (ROSC) was 5/8 (62.5%) in both groups; *p* = 1.00. Mean time to ROSC was similar in both groups; 6 minutes and 42 ± 65 s in the LMA group, and 6 min and 46 ± 51 s in the ETT group; *p* = 0.92. There was no difference in plasma epinephrine levels at baseline (LMA 0.7 ± 0.5 vs. ETT 0.8 ± 0.2 ng/mL; *p* = 0.88) and post-airway epinephrine administration (LMA 8.0 ± 3.1 vs. ETT 7.8 ± 4.6 ng/mL; *p* = 0.85).

**Conclusion:**

The use of LMA epinephrine for neonatal resuscitation is a suitable alternative to ETT epinephrine.

**Impact:**

- The Neonatal Resuscitation Program (NRP) recommends a dose of airway epinephrine to be administered via the endotracheal tube (ETT) while intravenous access is being established. Endotracheal intubation is a highly skilled procedure. Laryngeal mask airway (LMA) is as effective in delivering positive pressure ventilation (PPV) and requires less training to achieve adequate competency to secure a stable airway. We show that LMA is a similar method to deliver epinephrine during neonatal resuscitation in the ovine model.

## Introduction

Approximately 2.5 million infants die annually, and a quarter of these deaths are caused by birth asphyxia.^1^ Asphyxial cardiac arrest in neonates often requires PPV via an advanced airway such as an endotracheal tube (ETT) or laryngeal mask airway (LMA) when facemask ventilation fails or if chest compressions (CC) are required.^2,3^ Despite chest compressions, if heart rate (HR) remains <60 bpm, the NRP recommends administration of 1 ml/kg of epinephrine (0.1 mg/ml) via the airway until vascular access is obtained. Traditionally, airway epinephrine has been given via the ETT, but we propose the LMA as a potential alternative route.

ETT insertion in a newborn is a highly skilled procedure that requires considerable training and experience.^4^ Even resuscitators with advanced training sometimes require prolonged or multiple attempts to intubate the neonatal trachea successfully.^5^ One study showed the average time taken for LMA insertion (9.7 ± 3.25 s) to be significantly shorter than that of ETT insertion (18.08 ± 4.8 s) (*p* < 0.001).^6^ Some studies reported the LMA to be more effective than a facemask in delivering PPV in neonates.^7,8^ Current evidence from studies comparing the LMA to ETT have shown both modalities to have comparable ventilation parameters during PPV.^5,6,8,9^ LMA insertion requires less training and experience to achieve adequate competency to secure a stable airway.^8,9^ This is especially relevant to healthcare personnel attending deliveries with limited expertise in neonatal intubation. Additionally, the laryngeal mask airway provides positive pressure ventilation with low pressures in the respiratory passage and reduced air losses compared to an uncuffed endotracheal tube with the advantage of lower airway resistance, thus requiring significantly lower peak inspiratory pressures (PIP) to achieve similar tidal volumes.^10,11^ However, in spite of the advantages LMA provides, the efficacy of LMA epinephrine in the neonatal model of cardiac arrest has not been studied, to our knowledge. We hypothesize that LMA epinephrine is comparable to ETT epinephrine.

## Methods

### Ethics statement

The study protocol was approved by the Institutional Animal Care and Use Committee (IACUC) at the University of Buffalo. All procedures were consistent with the Animal Research: Reporting of In Vivo Experiments (ARRIVE) guidelines and were approved by the Institutional Animal Care and Use Committee at the State University of New York at Buffalo.^12^

### Animal preparation

A translational lamb model of asphyxial cardiac arrest is a well-established model to study the gas exchange, and systemic and pulmonary hemodynamics during neonatal resuscitation.^13^

**Fig. 1:**
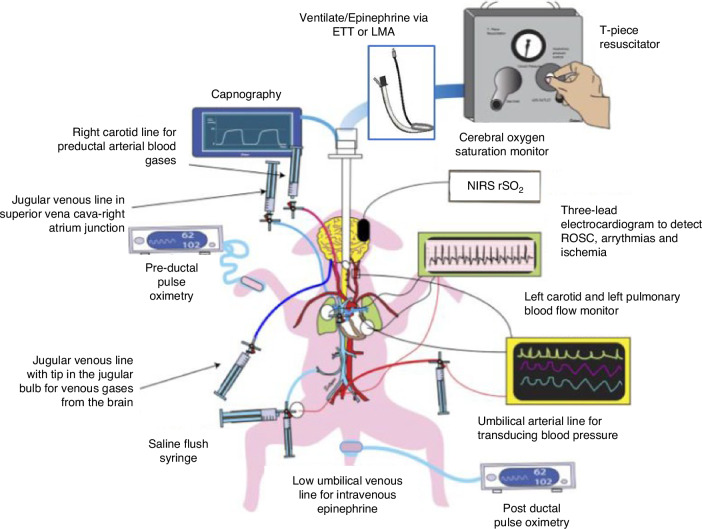
Instrumentation of near-term lamb model of cardiac arrest (Illustration by Dr. Satyan Lakshminrusimha).

Time-dated ewes (near-term 139–142 days of gestation) were fasted overnight for 12 h in preparation for anesthesia which is induced by using intravenous ketamine and diazepam. Ewes were then intubated with an 8-10 mm cuffed endotracheal tube and placed on a volume ventilator with a precision isoflurane vaporizer. Ventilation parameters for ewes include tidal volume of 650 mL, respiratory rate of 16-20 breaths/min, isoflurane 2–4%, and fraction of inspired oxygen (FiO_2_) at 1.00. Pulse oximetry was continuously monitored. Fetal lambs, anesthetized via placental transfer of maternal isoflurane were exteriorized via hysterotomy and intubated. Lung liquid was drained by gravity. Flow transducers were placed around the left carotid artery and left pulmonary artery as well as the ductus arteriosus. Catheters were placed in the right carotid artery for continuous blood pressure monitoring and arterial blood gas draws. Right jugular vein catheters were placed for fluid administration and venous blood gas draws. A single lumen 5 Fr catheter was placed in an umbilical vein and advanced 2 cm from the skin surface for IV epinephrine treatment (Fig. 1).

### Experimental protocol

This was a randomized controlled trial. Sixteen near-term lambs were randomized using a sealed envelope to LMA or ETT for ventilation and airway epinephrine administration. Resuscitators could not be blinded to the choice of alternative airway due to the nature of the intervention being studied (ETT vs. LMA). For the LMA group, we used LMA Supreme^TM^ (Laryngeal Mask Airway Co. Ltd, Jersey, UK) size 1, which was assessed to be the appropriate size for <5 kg lambs in a previous study in our lab.^5^

Cardiac arrest was induced by occlusion of both the umbilical cord and endotracheal tube. After cardiac arrest (defined by zero carotid artery flow and flat line on electrocardiogram tracing), umbilical cord was cut, and lamb was transferred to warmer and weighed on the bed-scale. The endotracheal tube was removed, placement of LMA or ETT was done based on randomization and positive pressure ventilation (PPV) was initiated after 5 min of cardiac arrest. A T-piece device with peak inspiratory pressure (PIP) of 35 cmH_2_O and PEEP of 5 cmH_2_O was used for PPV delivery. The fraction of inspired oxygen (FiO_2_) was set at 0.21 initially and after 30 s of effective ventilation oxygen was increased to 1.00 and chest compressions were initiated at the ratio of 3 compressions to 1 breath. PIP was titrated to maintain a targeted tidal volume of 7–9 ml/kg during resuscitation using respiratory function monitor. Of note, the lamb neonatal model has a higher amount of fetal lung fluid in comparison to the human neonate, and a higher tidal volume requirement (approximately 8 ml/kg).

**Fig. 2:**
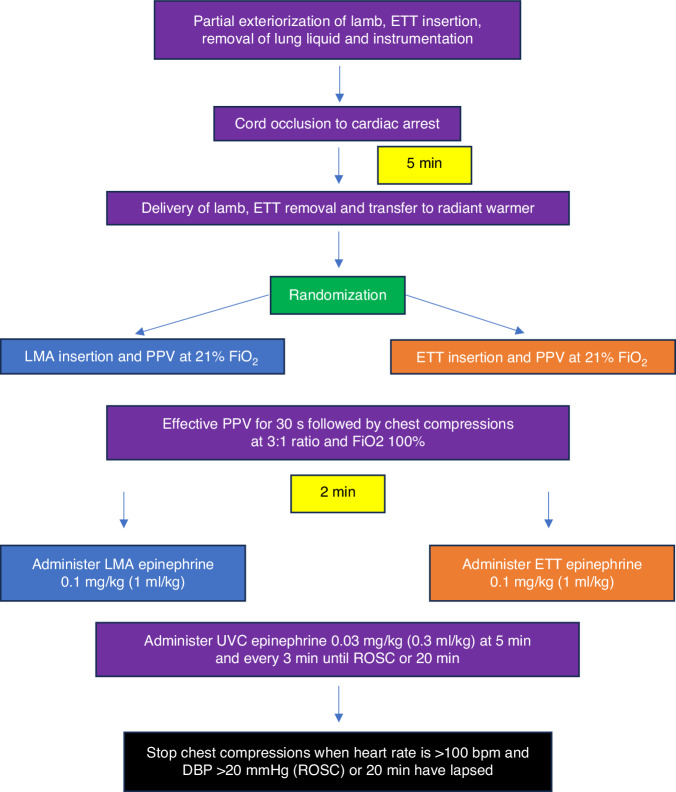
Flow diagram showing the experimental protocol. Near-term lambs were instrumented, asphyxiated to asystole and resuscitated per NRP guidelines. Lambs were randomized to epinephrine via the endotracheal tube or laryngeal mask airway groups. Airway epinephrine was administered 2 minutes after positive pressure ventilation and chest compressions were initiated.

An airway dose of epinephrine (0.1 mg/kg or 1 ml/kg; high dose epinephrine as currently recommended by the NRP) was administered using a syringe after 2 min from initiation of PPV. A dose of IV epinephrine (0.03 mg/kg or 0.3 ml/kg) was administered at 5 min of cardiac arrest via UVC followed by 3 mL of Normal Saline flush, then repeated every 3 min as needed. Lambs were resuscitated for 20 minutes or until ROSC and observed for 30 min post ROSC. ROSC was defined as a sustained heart rate ≥100 beats per minutes with a diastolic blood pressure of ≥20 mmHg (Fig. 2). NRP guidelines were followed post-ROSC for weaning oxygen and managing ventilation.

Blood from the carotid artery (0.2 ml) and the jugular vein (0.2 ml) was drawn simultaneously at baseline prior to resuscitation, every minute during resuscitation, at ROSC, at 1- and 2-min post ROSC, then every 5 min thereafter for arterial and venous blood gas analysis, and plasma epinephrine measurement until 30 min post ROSC. In non-ROSC lambs, blood was collected as above, then discontinued after the blood draw at 20 min.

Continuous hemodynamic measurements were recorded via Biopac software.

The following data was collected during resuscitation:

1. Systolic, diastolic, and mean blood pressure (mmHg).
2. Electrocardiogram (ECG) data during chest compressions, ROSC, and post-ROSC.
3. Carotid blood flow (ml/kg/min).
4. Pulmonary artery flow (ml/kg/min).

After resuscitation, the LMA/ETT were removed at sacrifice and rinsed in a 50 mL conical tube with 10 mL normal saline repeatedly flushing both inside and outside of the tubing. The sample was frozen at -80 degrees Celsius until an aliquot was measured by enzyme-linked immunosorbent assay (ELISA) (Eagle Bioscience). The result (ng/ml) was corrected using the total rinse volume to yield the total residual epinephrine (ng) remaining in the tubing.

### Sample size calculation

Previously, in this model we have shown the median (IQR) time to achieve ROSC was 6.85 min (6–9.1 min) in the LMA group and 7.50 min (5.33–18 min) in the ETT group.^5^ With a 5% level of significance, 80% power and equivalence defined as no more than 1 minute difference in time to ROSC between groups at any time, 16 lambs (8 in each group) were calculated to be required to demonstrate equivalence.

### Statistical analysis

Continuous data with a normal distribution such as trends in blood pressure and arterial flows was analyzed by ANOVA. Dichotomous data was analyzed with Fisher’s exact test using Stat view 4.0 and SPSS 21 software.

## Results

Baseline characteristics were similar in the two groups (Table 1).

**Table 1:** Baseline patient characteristics were similar between the two groups.

Variable	ETT (*n* = 8) Mean ± SD	LMA (*n* = 8) Mean ± SD	*P*-value
Gestation (days)	139.8 ± 0.89	139.6 ± 0.52	0.34
Birth weight (kg)	3.96 ± 0.62	4.26 ± 0.52	0.35
Gender (M:F)	3:5	5:3	0.32
Baseline pH (pre ETT occlusion)	7.21 ± 0.11	7.29 ± 0.09	0.19
pH after asphyxia	6.85 ± 0.11	6.89 ± 0.07	0.36
Baseline lactate (mmol/L)	5.8 ± 2.5	5.0 ± 2.5	0.56
Lactate level after asphyxia (mmol/L)	10.3 ± 4.2	8.5 ± 2.6	0.32
Lung fluid volume (mL)	86.5 ± 31.9	79.6 ± 20.6	0.62
Time to asystole (min)	13.67 ± 6.06	13.88 ± 4.85	0.94

### Return of spontaneous circulation

Five out of 8 (62.5%) lambs in both groups achieved ROSC (*p* = 1.00). Time to ROSC was similar in both groups, 6 min and 46 s in ETT versus 6 minutes and 42 seconds in the LMA group (*p* = 0.92) (Table 2).

**Table 2:** ROSC time and incidence was similar between the two groups.

	ETT (*n* = 8)	LMA (*n* = 8)	*P*-value
Incidence of ROSC, *n* (%)	5/8 (62.5%)	5/8 (62.5%)	1.00
Time to ROSC, Mean ± SD	6 min 46 s ± 51 s	6 min 42 s ± 65 s	0.92
Lambs that received airway Epinephrine, *n* (%)	8/8 (100%)	8/8 (100%)	1.00
ROSC lambs that received 1 dose of UVC Epinephrine, *n* (%)	5/5 (100%)	5/5 (100%)	1.00
ROSC lambs that received > 1 dose UVC Epinephrine, *n* (%)	1/5 (20%)	0/5 (0%)	0.46

All lambs received airway epinephrine 0.1 mg/kg (1 ml/kg) at 2 minutes of resuscitation. In the ETT group that achieved ROSC, 1 of 5 required 2 doses of IV epinephrine while the others achieved ROSC after the first dose. All five lambs that achieved ROSC in the LMA group only required one dose of IV epinephrine. None of the lambs in either group achieved ROSC after airway epinephrine alone.

### Systemic and pulmonary hemodynamics

**Fig. 3:**
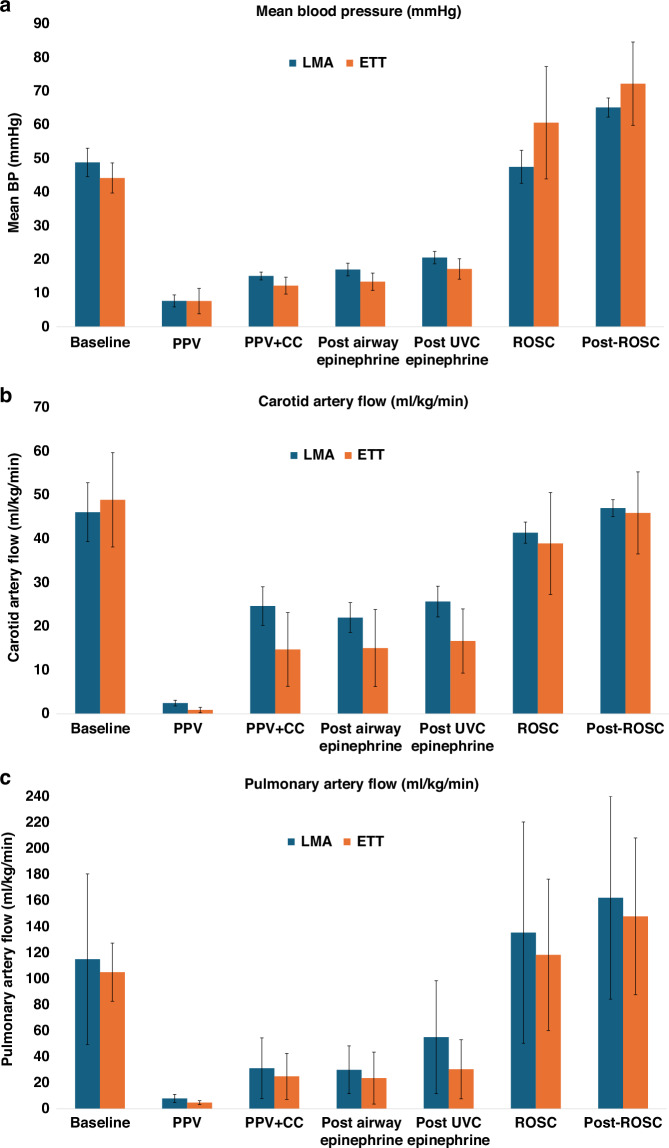
Systemic and pulmonary hemodynamic parameters. **a** Mean arterial blood pressure was similar between the two groups. **b** Carotid artery flow was similar between the two groups. **c** Pulmonary artery flow was similar between the two groups.

Following epinephrine administration via the airway, mean blood pressures (LMA 17 ± 5.3 mmHg vs. ETT 13.4 ± 2.6 mmHg; *p* = 0.13), carotid artery flow (LMA 21.9 ± 9.7 ml/kg/min vs. ETT 15 ± 8.8 ml/kg/min; *p* = 0.24) and pulmonary artery flow (LMA 30 ± 18.4 ml/kg/min vs. ETT 23.6 ± 20 ml/kg/min; *p* = 0.59) were not statistically different between the two groups. Mean blood pressure, carotid artery and pulmonary artery flows were similar throughout resuscitation (Fig. 3a, b, c).

### Gas exchange parameters

**Fig. 4:**
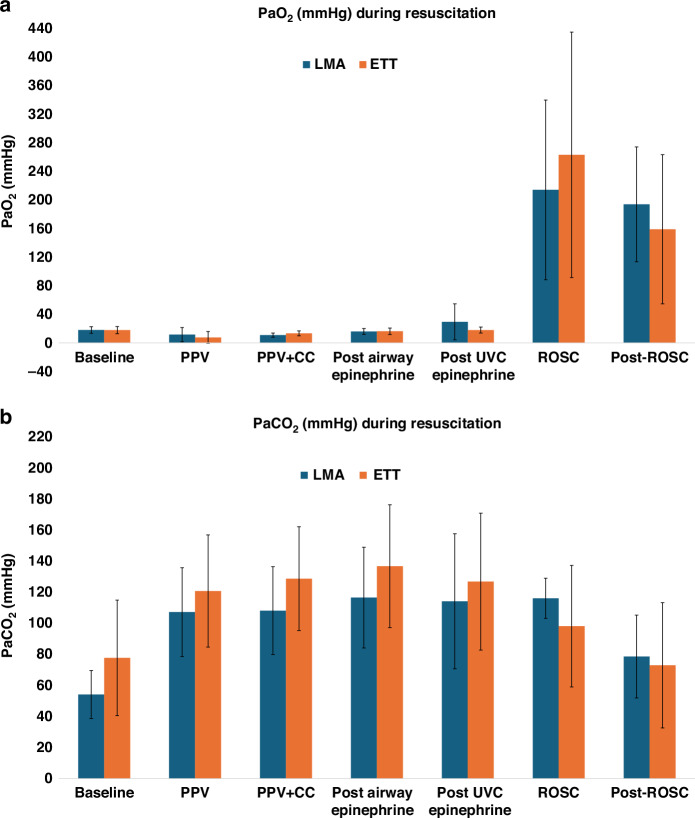
Gas exchange. **a** Partial pressure of arterial oxygen was similar between the two groups. **b** Partial pressure of arterial carbon dioxide was similar between the two groups.

The partial pressure of arterial oxygen (LMA 16 ± 4.1 mmHg vs. ETT 16.3 ± 4.5 mmHg; *p* = 0.89) (Fig. 4a), and partial pressure of arterial carbon dioxide (LMA 116 ± 35 mmHg vs. ETT 135 ± 40 mmHg; *p* = 0.32) (Fig. 4b) were similar after administration of airway epinephrine.

### Epinephrine levels

**Fig. 5:**
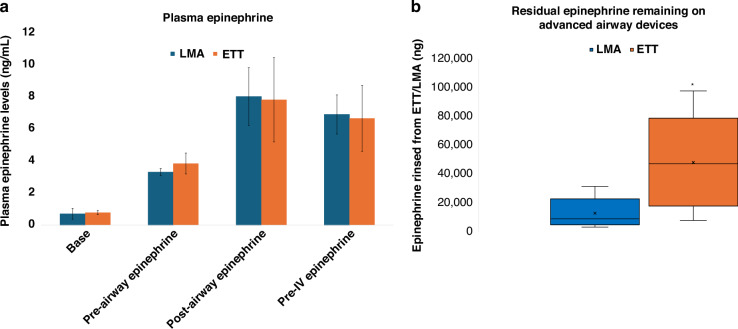
Epinephrine levels. **a** Plasma epinephrine levels after administration of airway epinephrine was similar between the two groups. **b** Residual epinephrine rinsed from ETT was significantly higher than residual epinephrine rinsed from LMA at the end of experiments. X: mean. Line inside boxes: median. Square edges: 1st and 3rd quartiles [interquartile range]. Whiskers: minimum and maximum values) (*p value < 0.05).

There was no statistically significant difference in plasma epinephrine levels post-airway epinephrine administration (LMA 8.0 ± 3.1 vs. ETT 7.8 ± 4.6 ng/mL; *p* = 0.85) (Fig. 5a).

The amounts of residual epinephrine rinsed from airway after resuscitation was significantly higher in ETT (48,220 ± 30,489 ng) than in LMA (12395 ± 9325 ng); *p = 0.015 (Fig. 5b).

## Discussion

To our knowledge, this is the first study evaluating efficacy of epinephrine via LMA in a neonatal cardiac arrest model. We concluded that there was no difference between ETT and LMA epinephrine administration during resuscitation on incidence or time to ROSC, mean blood pressure, carotid artery flow, pulmonary artery flow, tidal volume, or plasma epinephrine levels. The use of LMA epinephrine for neonatal resuscitation is comparable and a suitable alternative to ETT epinephrine while establishing an umbilical venous line.

Asphyxiated neonates receiving chest compressions have low systemic vascular resistance. During chest compressions, blood preferentially travels through the aorta into the vasodilated peripheral circulation instead of the narrower coronary arteries, following the path of least resistance. Coronary arteries receive their blood supply during diastole when the left ventricle relaxes, and coronary vascular resistance drops. At high doses, epinephrine exerts alpha-1 adrenergic effects that lead to potent vasoconstriction.^14^ Epinephrine improves coronary perfusion by increasing systemic vascular resistance and diastolic blood pressure, which in turn leads to blood flow into the coronary arteries during diastole. Reestablishing coronary perfusion is essential for achieving ROSC.^15^ The most optimal intravenous (IV) access in a scenario of asphyxial cardiac arrest in the neonate is placing an umbilical venous catheter (UVC). However, obtaining UVC access is a process that requires expertise and may take 5–8 min on average.^16^ Therefore, the NRP recommends the first dose of epinephrine to be administered via the ETT.^14^ Multiple studies in the past have shown surfactant administration via LMA to be as effective as ETT,^17–19^ but there is no data on epinephrine administration via LMA during resuscitation in neonates.

LMA insertion is an easy skill for healthcare providers to acquire and maintain, with the potential to improve newborn resuscitation in regions lacking neonatal expertise, both in the USA and globally. In developing countries, where 98% of the 7 million annual perinatal deaths occur, over 75% of neonatal resuscitations are performed by nurses, midwives, and traditional birth attendants.^20^ The First Breath Study showed that modified NRP training for such providers, excluding skills like intubation, chest compressions, and medication use, did not reduce early neonatal deaths. Training a skilled neonatal care workforce in rural, low-income areas is challenging. In this context, incorporating LMA and epinephrine administration via LMA as a routine, evidence-based approach could provide a sustainable, cost-effective alternative.^21^ LMA insertion can be taught quickly, providers can achieve proficiency after 15 min of manikin training, with successful insertion occurring within 5–10 s.^22,23^ Laryngeal mask ventilation is safe and effective for providers with limited endotracheal intubation skills.^9,24^

In our study, none of the neonatal lambs achieved ROSC after airway epinephrine, and all required IV epinephrine to achieve ROSC. Plasma epinephrine was 8.0 ± 3.1 ng/mL for the LMA group and 7.8 ± 4.6 ng/mL for the ETT group after airway epinephrine was administered. ROSC in the lambs was only achieved when plasma epinephrine was 362 ± 192 ng/mL in both groups. In spite of using high-dose airway epinephrine, plasma epinephrine did not reach the levels required to achieve ROSC.

Previous studies in the adult swine^25^ and piglet^26^ models showed more significant increases in plasma epinephrine levels after ETT epinephrine was administered compared to our study. However, both those models did not have retained lung fluids. The piglet study was done on 2–4-day old piglets that were born vaginally after labor with minimal retained lung fluid, as compared to our neonatal ovine model where the airway epinephrine was administered soon after birth, and lambs were born via cesarian section with no preceding labor with estimated retained lung fluid volume of approximately 28–38 ml/kg.^27,28^

A recent study reported higher rates of ROSC in neonates that received an initial ETT dose of epinephrine followed by IV epinephrine (70.1%), compared to initial IV epinephrine without initial ETT epinephrine dose (58.3%).^29^

As for the differences observed in residual epinephrine left on ETT and LMA appliances, we speculate this was due to difference in length and diameter. The ETT is longer and narrower compared to the LMA. However, there was no clinical significance since plasma epinephrine levels were similar in both groups, possibly due to epinephrine leaking to the stomach when given via LMA.

The main limitation of our study is that we did not reach the sample size of 8 surviving lambs in both groups, required to assess a difference in our primary outcome (time to ROSC), as three lambs in each group did not achieve ROSC. Due to ethical concerns and financial constraints, the desired number of subjects could not be attained. For similar reasons, it would be very difficult to power such a study to assess differences in plasma epinephrine levels as it would require a considerably larger sample size.

Another limitation in our study is the difference in species. Newborns requiring chest compressions during resuscitation at delivery is rare. It is estimated to occur in 0.08% of all term and near-term deliveries.^30^ The rarity of the problem makes it difficult to conduct a clinical study to evaluate the efficacy of epinephrine through LMA during chest compression. Additionally, unlike clinical scenarios, these studies were done under a controlled environment with highly skilled resuscitators.

Our study is the first to show that LMA epinephrine may be as effective as ETT epinephrine in a neonatal ovine model. Inexperienced resuscitators can provide superior ventilation via the LMA when compared to facemask ventilation and would have a potential means to administer epinephrine when the ability to insert an endotracheal tube or UVC is lacking in resource-limited settings.

Despite the controversies surrounding the use of airway epinephrine, some studies suggest that the administration of airway epinephrine result in higher rates of ROSC after administration of IV epinephrine as compared to IV epinephrine alone. Our study was unfortunately underpowered due to some of our lambs not achieving ROSC, however, it suggests that epinephrine administered via the LMA may be as effective as ETT. Larger-scale clinical studies are needed to assess the true efficacy of airway epinephrine in neonates.

## Supplementary information

Author Checklist

IACUC approval

## Data Availability

The datasets generated during and/or analyzed during the current study are available from the corresponding author on reasonable request.
