# Correction: Efficacy of laryngeal mask epinephrine in neonatal resuscitation; an ovine study

**DOI:** 10.1038/s41390-025-04546-4

**Published:** 2025-10-20

**Authors:** Hamza Abbasi, Clariss Blanco, Arun Prasath, Mary Kasu, Sylvia Gugino, Justin Helman, Nicole Bradley, Lori Nielsen, Rocco A. Paluch, Praveen Chandrasekharan, Munmun Rawat

**Affiliations:** 1 John R. Oishei Children’s Hospital, Neonatology Department, Buffalo, NY USA; 2 State University of New York at Buffalo, Buffalo, NY USA; 3 NYC Health and Hospitals/Harlem, New York, NY USA; 4 University of Texas Southwestern, Dallas, TX USA; 5 University of Maryland Medical Center, Baltimore, MD USA

Correction to: *Pediatric Research* 10.1038/s41390-025-04070-5, published online 12 May 2025

In the sentence beginning ‘We show that LMA...’ in this article, the term ‘non-inferior’ should have read ‘similar’.

In the sentence beginning ‘This was a randomized...’ in this article, the term ‘non-inferiority’ has been redacted.

The original article has been corrected.

