# Supplementary material for: Efficacy of laryngeal mask epinephrine in neonatal resuscitation; an ovine study
Source: Pediatr Res. 2025 May 12;99(6):2345–51. doi: 10.1038/s41390-025-04070-5 (PMC13433248; doi:10.1038/s41390-025-04070-5)
Supplement: Supplementary file 1 [file 41390_2025_4070_MOESM1_ESM.pdf]

NOTE: Please save this file locally before filling in the table, DO NOT work on the file within your internet browser as changes will not be saved. Adobe Acrobat Reader (available free here) is recommended for completion.

The ARRIVE guidelines 2.0: author checklist

The ARRIVE Essential 10

These items are the basic minimum to include in a manuscript. Without this information, readers and reviewers cannot assess the reliability of the findings.

Item		Recommendation	Section/line number, or reason for not reporting
**Study design**	1	For each experiment, provide brief details of study design including:	
		a. The groups being compared, including control groups. If no control group has been used, the rationale should be stated.	randomized controlled trial
		b. The experimental unit (e.g. a single animal, litter, or cage of animals).	16 near term lambs
**Sample size**	2	a. Specify the exact number of experimental units allocated to each group, and the total number in each experiment. Also indicate the total number of animals used.	8 in each group
		b. Explain how the sample size was decided. Provide details of any *a priori* sample size calculation, if done.	With 0.8 standard deviation in ROSC, 16 lambs (8 per group) are required to demonstrate that the lower limit of a one-sided 95% confidence interval will be below the non inferiority limit of 1. This
**Inclusion and exclusion criteria**	3	a. Describe any criteria used for including and excluding animals (or experimental units) during the experiment, and data points during the analysis. Specify if these criteria were established *a priori*. If no criteria were set, state this explicitly.	NA
		b. For each experimental group, report any animals, experimental units or data points not included in the analysis and explain why. If there were no exclusions, state so.	No exclusions
		c. For each analysis, report the exact value of *n* in each experimental group.	N of 16, 8 in each group
**Randomisation**	4	a. State whether randomisation was used to allocate experimental units to control and treatment groups. If done, provide the method used to generate the randomisation sequence.	Sixteen near-term lambs were randomized using a sealed envelope to LMA or ETT for ventilation
		b. Describe the strategy used to minimise potential confounders such as the order of treatments and measurements, or animal/cage location. If confounders were not controlled, state this explicitly.	NA
**Blinding**	5	Describe who was aware of the group allocation at the different stages of the experiment (during the allocation, the conduct of the experiment, the outcome assessment, and the data analysis).	Researchers not blinded
**Outcome measures**	6	a. Clearly define all outcome measures assessed (e.g. cell death, molecular markers, or behavioural changes).	hemodynamics/vital signs, plasma epinephrine levels, blood gases, residual epineprhine on LMA and ETT devices
		b. For hypothesis-testing studies, specify the primary outcome measure, i.e. the outcome measure that was used to determine the sample size.	ROSC incidence and time to ROSC
**Statistical methods**	7	a. Provide details of the statistical methods used for each analysis, including software used.	Continuous data with a normal distribution such as trends in blood pressure and arterial flows was analyzed by ANOVA. Dichotomous data was analyzed with Fisher's exact test using Stat view 4.0 and
		b. Describe any methods used to assess whether the data met the assumptions of the statistical approach, and what was done if the assumptions were not met.	NA
**Experimental animals**	8	a. Provide species-appropriate details of the animals used, including species, strain and substrain, sex, age or developmental stage, and, if relevant, weight.	data provided in table 1
		b. Provide further relevant information on the provenance of animals, health/immune status, genetic modification status, genotype, and any previous procedures.	NA
**Experimental procedures**	9	For each experimental group, including controls, describe the procedures in enough detail to allow others to replicate them, including:	
		a. What was done, how it was done and what was used.	NA
		b. When and how often.	NA
		c. Where (including detail of any acclimatisation periods).	NA
		d. Why (provide rationale for procedures).	NA
**Results**	10	For each experiment conducted, including independent replications, report:	
		a. Summary/descriptive statistics for each experimental group, with a measure of variability where applicable (e.g. mean and SD, or median and range).	Available in manuscript
		b. If applicable, the effect size with a confidence interval.	Available in manuscript
