# Supplementary material for: Efficacy of laryngeal mask epinephrine in neonatal resuscitation; an ovine study
Source: Pediatr Res. 2025 May 12;99(6):2345–51. doi: 10.1038/s41390-025-04070-5 (PMC13433248; doi:10.1038/s41390-025-04070-5)
Supplement: Supplementary file 2 [file 41390_2025_4070_MOESM2_ESM.pdf]

University at Buffalo Institutional Animal Care and Use Committee (IACUC)
Office of Research Compliance | Clinical and Translational Research Center Room 5018
875 Ellicott St. | Buffalo, NY 14203

APPROVAL OF SUBMISSION

May 2, 2024

PRAVEEN CHANDRASEKHARAN
Oishei Children's Hospital of Buffalo
BUFFALO, NY 14222-0000

(716) 323 0260
pkchandr@buffalo.edu

Dear PRAVEEN CHANDRASEKHARAN:

The IACUC reviewed the following submission:

Type of Review:	Triennial Review
Title of Protocol:	Triennial Review for PED10085N
Investigator:	PRAVEEN CHANDRASEKHARAN
IACUC ID:	TR202400010
Funding:	None
Documents Reviewed:	• Dept Chair letter of scientific merit 3-15-2024.pdf<br>• Newborn Arrest Timeline.xlsx<br>• Bradycardia Timeline.xlsx<br>• Arrest Experiment Timeline<br>• Considerations on the Use of Neonatal and Pediatric Resuscitation Guidelines for Hospitalized Neonates and Infants: On Behalf of the American Heart Association Emergency Cardiovascular Care Committee and the American Academy of Pediatrics<br>• Emergency Contact

The Institutional Animal Care and Use Committee (IACUC) has reviewed and approved your submission for the project referenced above for a period ending on 5/1/2027. This approval is good only for the stated protocol and animals specified on this application form. A triennial application will need to be submitted at least 60 days prior to the expiration date above.

If your project at any time utilizes biohazardous or radioisotopes agents, use and purchasing of animals is contingent upon the completion of the proper training of your staff and, if necessary, the staff of the Laboratory Animal Facility (LAF) on the handling of the agent. Contact the Laboratory Animal Facility Manager, at 829-6826 before use of these agents. Please note that training of laboratory personnel is an annual requirement.

If you need help with Click please don't hesitate to contact our office.

Sincerely,
ROSEMARY DZIAK

Chair, Institutional Animal Care and Use Committee (IACUC)
